# The case for humour: Moving one step further

**DOI:** 10.4103/0019-5545.31566

**Published:** 2006

**Authors:** Chittaranjan Andrade

**Affiliations:** * Professor in Psychopharmacology, National Institute of Mental Health and Neurosciences, Bangalore 560029, Karnataka; e-mail: andrade@nimhans.kar.nic.in, andradec@gmail.com

Swaminath^1^ has cogently illustrated the use and value of humour in clinical interactions. Humour is indeed an important and much neglected tool; we could do with a lot more of it not only in the consulting room but also in the entire world in which we live.

Having said so, I wish to introduce a caveat. Few are artists or sculptors from the moment they begin to practice their art; most will need much instruction, and many will continue to fail with their brush or chisel no matter how long they train. Applying this metaphor, not every psychiatrist will have the personality or the talent to successfully use humour as a tool with his clients; the skill will necessarily need to be developed for use by the right person and in the right context, lest the attempts end on a negative note.

Moving on to a matter not addressed by Swaminath: that of the need for humour in academia. Clinicians, researchers, and editors who read, write in, or prepare journals have one attribute in common: they are all humans. Assuming that humour is a continuous variable that is normally distributed in the general population, and assuming that an interest in journals is not a confounding factor, a quick reference to statistical tables will show that more than four-fifths of journal readers will have humour levels that are within one standard deviation of the mean, or greater.

So, should there be more humour in scientific literature? I believe that there should. Journals would then become more interesting and more likely to be read, and readers would realize that even researchers and journal editors have a human side.

Scientists and dons have for long exhibited their wit; for example *Alice's adventures in wonderland* and *Through the looking glass* are, after the Bible and the works of Shakespeare, among the most quoted works in the world; these were written by the 19th century Oxford mathematician Charles Lutwidge Dodgson under the pen name Lewis Carroll.

The physical sciences have their humorists. For example, George Gamow^2^ discussed the Big Bang theory in a paper authored by his student Alpher, a colleague Bethe, and himself. Bethe did not actually participate in the work nor was informed that he was receiving a guest authorship in the paper. Gamow later confessed that he had included Bethe because he considered it only fitting for a paper on the origins of the universe to be authored by the first three letters of the Greek alphabet.

General medical journals have their humorists, too. For example, the *British Medical Journal* often publishes offbeat articles, especially in its Christmas issue, each year. Two studies that were particularly interesting were those of Leibovici^3^ and Smith and Pell.^4^ Leibovici conducted a randomized controlled study in which he said a short prayer for patients who had already been treated for septicaemia years earlier; he found that patients who were prayed for had been discharged significantly earlier than those who had not.^3^ Smith and Pell described a systematic review and meta-analysis of trials evaluating the usefulness of parachutes in the prevention of death due to free fall; finding no study which met their search criteria, they suggested that people who scorn observational data and believe only in evidence-based medicine should participate in randomized, double-blind, placebo-controlled, crossover trials of the efficacy of the parachute.^4^

In every issue, the *BMJ* also carries fillers, anecdotes and other interesting articles that are frequently humorous in content.^5^ The *Lancet* used to carry a regular humorous column titled Jabs and Jibes; regrettably, this column has not appeared for long. Other medical and specialty journals such as *JAMA* and *Neurology* include poetry, real-life story, and other columns, which, unfortunately, are sometimes tragicomic.

Psychiatry journals have not lagged behind. *Biological Psychiatry,* for example, once carried an April Fool editorial;^6^ the *BMJ,* itself, has often carried April Fool articles and responses thereto.^7^–^9^ Serious researchers in psychiatry have investigated semi-serious subjects and have published their results in high impact journals.^10^,^11^ Specialty journals in psychiatry have published articles that border on the spoof.^12^,^13^ The correspondence columns of most general psychiatry journals, too, are no strangers to letters in lighter vein.

In conclusion, I believe that, with the recognition of the importance of the views of Swaminath,^1^ and with the introduction of articles that are lighter in vein, the *Indian Journal of Psychiatry* will pass a new milestone in its history, and further improve its ratings and readability.
